# Vitamin K2 improves proliferation and migration of bovine skeletal muscle cells *in vitro*

**DOI:** 10.1371/journal.pone.0195432

**Published:** 2018-04-04

**Authors:** Sissel Beate Rønning, Mona Elisabeth Pedersen, Ragnhild Stenberg Berg, Bente Kirkhus, Rune Rødbotten

**Affiliations:** Nofima AS, Norwegian Institute of Food, Fisheries and Aquaculture Research, Ås, Norway; University of Patras, GREECE

## Abstract

Skeletal muscle function is highly dependent on the ability to regenerate, however, during ageing or disease, the proliferative capacity is reduced, leading to loss of muscle function. We have previously demonstrated the presence of vitamin K2 in bovine skeletal muscles, but whether vitamin K has a role in muscle regulation and function is unknown. In this study, we used primary bovine skeletal muscle cells, cultured in monolayers *in vitro*, to assess a potential effect of vitamin K2 (MK-4) during myogenesis of muscle cells. Cell viability experiments demonstrate that the amount of ATP produced by the cells was unchanged when MK-4 was added, indicating viable cells. Cytotoxicity analysis show that MK-4 reduced the lactate dehydrogenase (LDH) released into the media, suggesting that MK-4 was beneficial to the muscle cells. Cell migration, proliferation and differentiation was characterised after MK-4 incubation using wound scratch analysis, immunocytochemistry and real-time PCR analysis. Adding MK-4 to the cells led to an increased muscle proliferation, increased gene expression of the myogenic transcription factor *myod* as well as increased cell migration. In addition, we observed a reduction in the fusion index and relative gene expression of muscle differentiation markers, with fewer complex myotubes formed in MK-4 stimulated cells compared to control cells, indicating that the MK-4 plays a significant role during the early phases of muscle proliferation. Likewise, we see the same pattern for the relative gene expression of collagen 1A, showing increased gene expression in proliferating cells, and reduced expression in differentiating cells. Our results also suggest that MK-4 incubation affect low density lipoprotein receptor-related protein 1 (LRP1) and the low-density lipoprotein receptor (LDLR) with a peak in gene expression after 45 min of MK-4 incubation. Altogether, our experiments show that MK-4 has a positive effect on muscle cell migration and proliferation, which are two important steps during early myogenesis.

## Introduction

Vitamin K is a fat-soluble vitamin present in many foods including vegetables, fish, meat, cheese and eggs. This is not a single molecule, rather a group of closely related derivates with a 2-methyl-1,4-naphthoquinone structure as its common framework. The K vitamins differ from each other by their length and saturation of their isoprenoid side chain. Vitamin K2 is made up of a group of menaquinones (MKs), in which MK-4 has the shortest isoprenic side chain. Menaquinones are present in various amounts in animal (such as meat and cheese) and plant-based fermented food (e.g. fermented cabbage)[1]. Almost all the menaquinones, in particular the long-chain menaquinones (i.e. MK-7), are also produced by bacteria in the human gut. Vitamin K1 (phylloquinone) found primarily in plant foods can be converted to MK-4 following oral and enteral administration, and this conversion process does not involve bacterial action[2]. Phylloquinone is tightly bound to chloroplasts, so it is suggested to be less bioavailable than the menaquinones derived from animal sources, which are consumed in fatty food matrices that potentially improve bioavailability. The bioavailability seems to be closely associated with the length of the side chain and lipophilicity [2–4]. In a study comparing *in vivo* properties of vitamin K1 and MK-7, the latter was more effective in catalysing osteocalcin carboxylation in bone and counteracting coumarin anticoagulants in the liver. According to the authors, an explanation could be that menaquinones, such as MK-7, have a much longer half-life [5].

Vitamin K was first recognized for its vital function in coagulation of blood, and is important for bone formation, soft-tissue calcification, and regulation of calcium content, cell growth and apoptosis [1, 6, 7]. Vitamin K has also been shown to have anti proliferative effects on several types of cancer cells [8, 9]. Bone mineral density and subsequent bone strength is determined by a fine-tuned balance between the activity of osteoblasts (bone production) and osteoclasts (bone resorption), both regulated by vitamin K2 [10]. There is a close relationship between bone and muscle, these two tissues share common regulatory signalling pathways, and numerous studies indicate that an increase in bone mineral density and reduced bone fraction risk is associated with an increase in muscle mass [11, 12]. The skeletal muscles account for a large part of the human body mass and are mainly composed of post-mitotic, multinucleated muscle fibres. The skeletal muscle comprises more than 600 individual muscles, important for movement and structure of the major metabolic organs. Skeletal muscle function is highly dependent on its ability to regenerate. Fifty years ago Mauro first suggested that satellite cells were involved in the skeletal muscle regeneration[13]. Since the first discovery of these cells, numerous reports have identified these stem cells as primary contributors to the postnatal growth, maintenance and repair of skeletal muscles. The satellite cells are normally quiescent in the adult muscle before they become activated upon exercise, injury or disease. These cells have a remarkable ability to self-renew, expand, migrate, proliferate and undergo myogenic differentiation to fuse and restore damaged muscle[14]. Cell migration is crucial for cell-cell adhesion, and therefore important for muscle proliferation, muscle differentiation as well as the formation and growth of myotubes *in vitro* [15]. Identification of molecules that regulate cell migration might reveal potential molecular targets for improving muscle regeneration. Furthermore, muscle cells exhibit different migratory behaviour throughout myogenesis *in vitro*[15]. During ageing or disease, the proliferative capacity is reduced, leading to loss of muscle function. Therefore, it is important to identify components that can help stimulate muscle proliferation, and as such improve muscle maintenance. We have previously shown that MK-4 is present in varying amounts in different bovine muscles and breeds[16]. However, few studies exist on a possible role of vitamin K2 in muscle regulation and function. In this study, we aimed to assess a potential effect of vitamin K2 in skeletal muscle using an *in vitro* model of bovine skeletal muscle cells.

## Materials and methods

### Vitamin K2

100 mg Menaquinone, MK-4 (Sigma Aldrich), was dissolved in 2.25 ml absolute Ethanol (EtOH).

### Bovine primary skeletal muscle cell isolation

Bovine primary skeletal muscle satellite cells were isolated as described previously [17]. The bovine primary skeletal muscle cells were obtained using fresh muscle samples from *Longissimus thoracis* (beef sirloin) from animals slaughtered at a commercial, industrial abattoir (Nortura AS, Rudshøgda, Norway). Animal Procedure approval was not required according to the Norwegian Food Safety Authority and the Norwegian Law. Animals of the same age (young animals), gender (bulls) and breed (Norwegian Red) were used for the muscle cell isolation. In brief, we digested small muscle pieces of ~ 1 g at 1h with 70 rpm shaking in 10 ml DMEM without FBS with 0.72 mg/ml collagenase at 37°C. The cells were further dissociated from the surrounding tissue by three treatments (of 25 min each) with 0.05% trypsin/EDTA. We added 10% FBS after each treatment to inactivate the trypsin, then we pooled the harvested cells. For removal of fast-adhering fibroblasts from the primary muscle cell cultures, the cells were placed in un-coated cell flasks for 1h at 37°C. This allowed the fibroblasts to adhere to the plastic. The non-adhering muscle cells were then collected and further seeded onto 25 cm^2^ Entactin-Collagen-Laminin (ECL)—coated culture flasks until 50% confluence. The isolated muscle cells were then collected, transferred onto 75 cm^2^ ECL-coated culture flasks until 70–80% confluence, and then stored in freezing media (1.5 ml growth media containing 8% dimethyl sulfoxide (DMSO) and 1 ml FBS) in liquid nitrogen until further use. All experiments were performed in second or third passage using proliferating cells. A characterization assay to confirm the purity and proliferation potential of myogenic cells was done on a routine basis after each cell isolation, staining for the well-known muscle marker neuron cell adhesion molecule (NCAM) and the proliferation marker myoD (S1 Fig) [18]. Normally the isolation procedures left us with a cell population of more than 90% myogenic cells. Previous characterisation of the primary muscle cells demonstrate a capacity to proliferate and differentiate [17].

### Cell culture and treatment

MK-4 was dissolved in absolute EtOH before addition to the cell culture medium (end concentration of EtOH was 0.4%). 0.4% EtOH was added to control cells. Tissue culture coverslips (Menzel-Gläser, Braunschweig, Germany), 96- and 6- well plates (BD Falcon, Franklin Lakes, NJ, USA), or cell culture flasks (VWR, West Chester, PA, USA) were coated with 3 μl/cm^2^ Entactin-Collagen IV-Laminin (1 mg/ml, Millipore, Billerica, MA, USA). Subsequently the coated surfaces were washed twice with PBS before cell culturing. Cells were plated at the following seeding densities: 3000 cells / well for 96-well micro plates, 20 000 cells/well for coverslips placed in 24 well plates, and 50 000 cells/well in 6-well plates. The primary cells were grown in Dulbecco’s modified Eagles’ medium (DMEM) with L-glutamine (2 mM), 2% FBS, 2% Ultroser G serum, P/S (10 000 units/ml), and Amphotericin B (250 μg/ml). The cells were cultivated in 2% FBS together with Ultroser G serum. Ultroser is a serum substitute that replaces the use of FBS for cell growth, and as such reduces the lot to lot serum variability often observed using FBS. Ultroser G serum substitute has a concentration five times higher than foetal calf serum (i.e. 2% of reconstituted Ultroser G serum substitute is equivalent to 10% foetal calf serum in the basal medium). Twenty-four hours after seeding (day 1), the cells were treated with MK-4, specific concentrations and time lengths are indicated in each figure legend. The concentrations chosen in this study reflect the varying levels found in related work (i.e. the effect of vitamin K on osteoclast differentiation and the effect of vitamin D on muscle proliferation/differentiation) [19, 20]. Then the cells were incubated with MK-4 or vehicle (control cells) for 3 days before proliferation analysis. For differentiation studies, proliferating cells (3 days), pre-incubated with or without MK-4, were washed with PBS and left in differentiation medium with or without MK-4 (DMEM, 2% FBS, P/S, Amphotericin B and 25 pmol Insulin) for 3 subsequent days to induce myogenesis.

### Cell viability, proliferation and cytotoxicity assay

These experiments were conducted for 72 hours on proliferating cells. The cells were seeded out in 96 well micro- plates (3000 cells/well) as recommended. The experiments were performed with at least three independent cell culture seeding experiments performed in triplicates. Cell viability was determined with CellTiter-Glo Luminescent Cell Viability Assay (Promega) with a cut-off value of 10% nonviable cells. Luminescence was detected using Glomax96 Microplate Luminometer (Promega). Cytotoxicity was measured as Lactate dehydrogenase (LDH) release into the cell media and was performed according to protocol (cat. no. 11 644 793 001, Roche Applied Science, Mannheim, Germany). Incubation with 2% Triton X-100 for 2h was used as a positive control. Cell proliferation was measured using CyQUANT cell proliferation assay (Invitrogen, Carlsbad, CA, US).

### In vitro scratch cell migration assay

Cell migration rates were assessed using a scratch wound healing assay [21]. Muscle cells were seeded out in 6-well plates and cultured until confluence. A scratch was then introduced to the cell monolayers using a 200 μl sterile pipette tip. To obtain the same field during the image acquisition, we created markings close to scratch that we used as a reference point. Cells were washed, and then incubated in fresh medium containing either 10 μM MK-4 or control (vehicle EtOH only) for 6h. The time frame of incubation was chosen to allow the cells to have the fastest migration conditions. Longer incubation times led to complete closure of the scratch. Images were then systematically obtained using Leica DMIL LED Light microscopy. Pictures were obtained with a reference mark outside the capture image field but within the eye-piece field of view. The distance between wound edges were manually measured for ten points of the cell migration edges on each image, and the values were used to calculate the percent gap closure at 6h relative to that at 0h. The experiments were performed with at least three independent cell culture experiments and a minimum of three images were quantified for each cell culture experiment.

### Fluorescence microscopy

Cells were grown on coated coverslips (Assistent, Sondheim/Rhön, Germany), washed in PBS twice and fixed in ice-cold ethanol for 15 min at -20°C. The cells were washed three times in PBS and blocked with 5% dry defatted milk for 30 min before incubation with the primary antibody for 1h. Subsequent incubation with secondary antibodies and Hoechst was performed for 30 min before mounting using the Dako fluorescent mounting medium (Glostrup, Denmark). The cells were examined by Fluorescence microscopy analysis (ZEISS Axio Observer Z1 microscope), and images were processed using Adobe Photoshop CS3. Rabbit anti-myod (MAB3876) was from Santa Cruz Biotechnologies Inc. (Santa Cruz, CA, USA), and rabbit anti-desmin (ab8592) was from Abcam (Cambridge, UK). Mouse anti-NCAM (5.1H11) was from Developmental Studies Hybridoma Bank (Iowa city, IA, USA). Hoechst (Hoechst 33342, 10 μg/ml), Alexa 488-conjugated goat anti-mouse and Alexa 546-conjugated goat anti-rabbit were from Thermo Fisher Scientific (Waltham, MA, USA). To quantify NCAM+/- myoblasts, immunostained cells were counted manually. The pictures were chosen randomly from at least three regions from each well to ensure a non-biased quantification. The fusion index (FI, i.e. the number of cells with more than 3 nuclei) was calculated from three independent cell culture experiments and scored from at least four randomly chosen regions containing nuclei and myotubes stained with anti-desmin. To quantify the number of myotube formations, at least six representative images per sample was scored. For each region the number of nuclei incorporated in myotubes and the total number of nuclei was scored. The fusion index was calculated as the percentage of total nuclei incorporated in myotubes.

### RNA extraction and real-time PCR

Cells were incubated in fresh medium containing either 10 μM MK-4 or control (vehicle EtOH only) for the indicated time points. Cells were lysed and further purified using RNeasy minikit including DNase treatment according to the manufacturer’s protocol (Qiagen, Hilden, Germany). cDNA was generated from ~400 ng mRNA using TaqMan^®^ Reverse Transcription Reagents (Invitrogen, Carlsbad, CA, USA) according to the manufacturer’s protocol. The cDNA was diluted four times before aliquots (in duplicates) were subjected to real-time PCR analysis using an ABI Prism 7700 Sequence Detection system (Applied Biosystem, UK) for experiments with proliferating cells, which was later replaced with QuantStudio 5 Real-Time PCR System (Applied Biosystem, UK) for the differentiation experiments and immediate uptake experiments. Amplification of cDNA by 40 two-step cycles (15 sec at 95°C for denaturation of DNA, 1 min at 60°C for primer annealing and extension) was performed, and cycle threshold (Ct) values were obtained graphically (Applied Biosystem, Sequence Detection System, Software version 2.2). Applied Biosystem’s primer/probe assays were used in this study: Bt04301299_m1 (LDLR), Bt03817622_g1 (LRP10), Bt03230910_m1 (DCN), Bt03225329_g1 (Col1A1), Bt033254509_g1 (Desmin), Bt03244740_m1 (myoD), Bt03258928_m1 (Myogenin), Bt04290445_gH (Myh8), Bt03223794_g1 (Elongation factor 1). Primers and probes of Syndecan-4, Integrin B1, β-actin [22] and Syndecan-1; Fwd. (TCTGCCTGTGTCCATGAACTTG), Rev. (TCTTCCCCAGCCTGAGACAT), probe (CCATGGCCTGGGCGACTACCATACT). Primers and probes were designed using the Primer Express Program (Applied Biosystems). Gene expression of the samples was normalized against the average value of Elongation factor 1 and β-actin, and ΔCt values were calculated according to the MIQE guidelines [23]. PCR efficiency and melting point analysis were performed on all targets. Comparison of the relative gene expression (fold change) between control (vehicle EtOH only) and treated cells (10 μM MK-4) was derived by using the comparative Ct method. In short, values were generated by subtracting ΔCt values between two samples which gives a ΔΔCt value. The relative gene expression (fold change) was then calculated by the formula 2-^ΔΔCt^ [24]. The efficiency of each set of primers was always higher than 96%. The real-time PCR was performed with two technical replicates in at least three independent cell culture experiments seeded out in duplicates.

### Statistical analysis

Significant variance by treatments in comparison to the control sample was determined either by 1) one-way ANOVA using Dunnett’s multiple comparison test and 2) unpaired two-tailed t-test. Differences were considered significant at p<0.05. All statistical analysis was performed in Graph Pad Prism version 7.03 (GraphPadSoftware, La Jolla, CA, USA).

## Results and discussion

**Fig 1:**
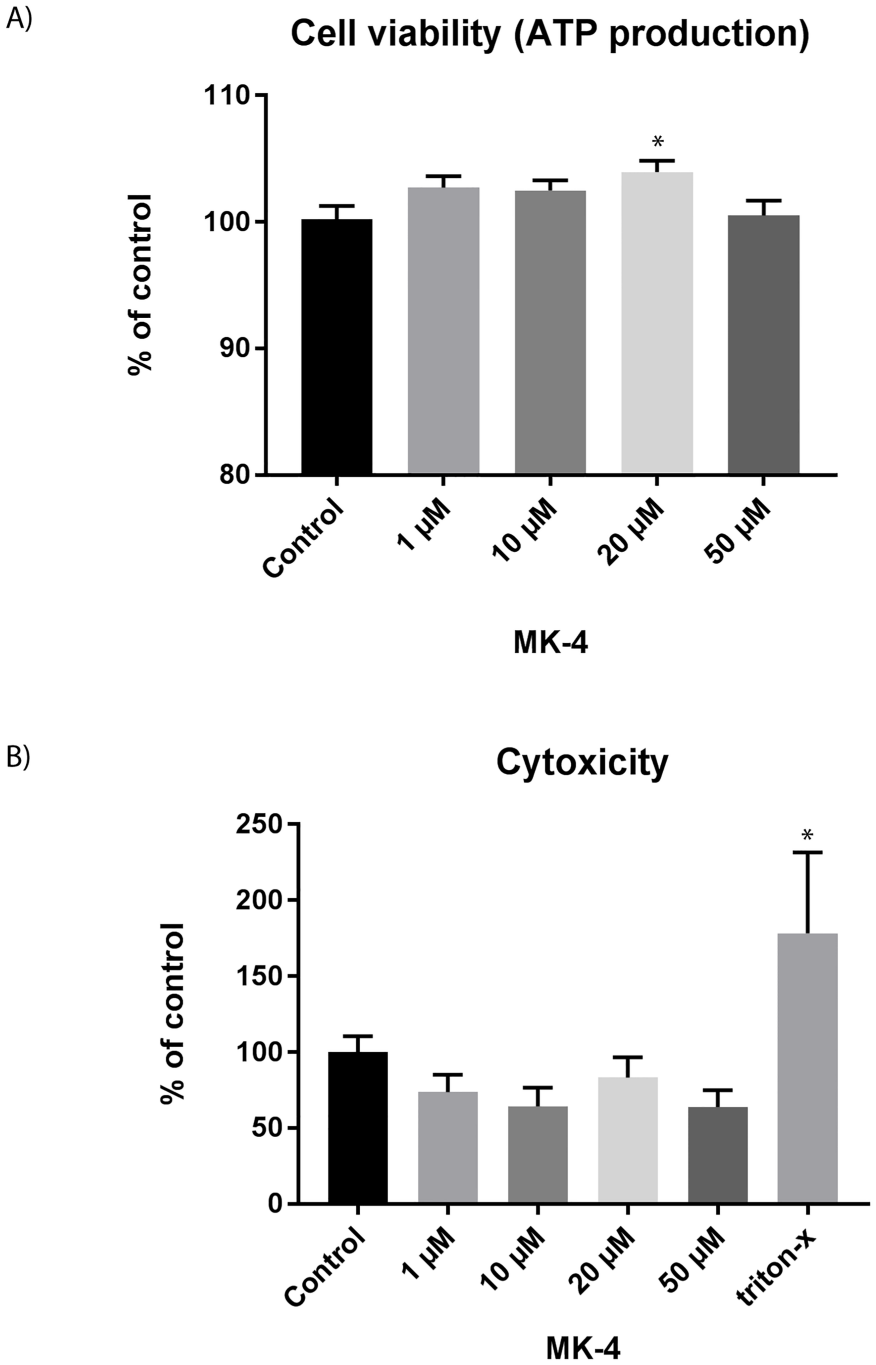
Viability and cytotoxicity upon MK-4 incubation for 72 h. A) Bars show the viability (amount of ATP present) of MK-4 treated cells compared to control cells (vehicle EtOH only). B). Bars show the amount of LDH release into the media in MK-4 treated cells compared to control cells (vehicle EtOH only). The data is presented as the average of at least three independent cell culture experiments seeded out in triplicates, ± SEM. Asterisk indicate significant differences (*p<0.05 in treated cells compared with control cells,) statistics assessed by one-way ANOVA with Dunnett’s multiple comparison test.

The presence of vitamin K2 in skeletal muscle [16] implies a possible role in muscle regulation and function. Our experiments demonstrate that the amount of ATP produced by the cells was unchanged when MK-4 was added to the cells, indicating that the muscle cells were viable and metabolically active (Fig 1A). It has previously been shown that MK-4 plays a function in mitochondria by speeding up the transfer of electrons, which results in more efficient ATP production [25]. In this way vitamin K2 may improve mitochondrial function and helps overcome mitochondrial defects. Skeletal muscle cells contain the highest mitochondrial content of any tissue in the body, and a decline in mitochondrial content and function is observed in muscle disease and during ageing. In fact, imbalance of skeletal muscle mitochondria is considered as one of the major factors contributing to the age-related loss of muscle mass and strength (sarcopenia) [26]. The enzyme lactate dehydrogenase (LDH) is found in all tissues and is normally released during tissue damage. LDH is therefore often used as a marker of muscle pathology and sarcopenia [27]. Our experiments showed that MK-4 slightly reduced the LDH release into the media (Fig 1B), suggesting that MK-4 was beneficial to the muscle cells. As such, our findings suggest MK-4 to have a positive impact on muscle function.

**Fig 2:**
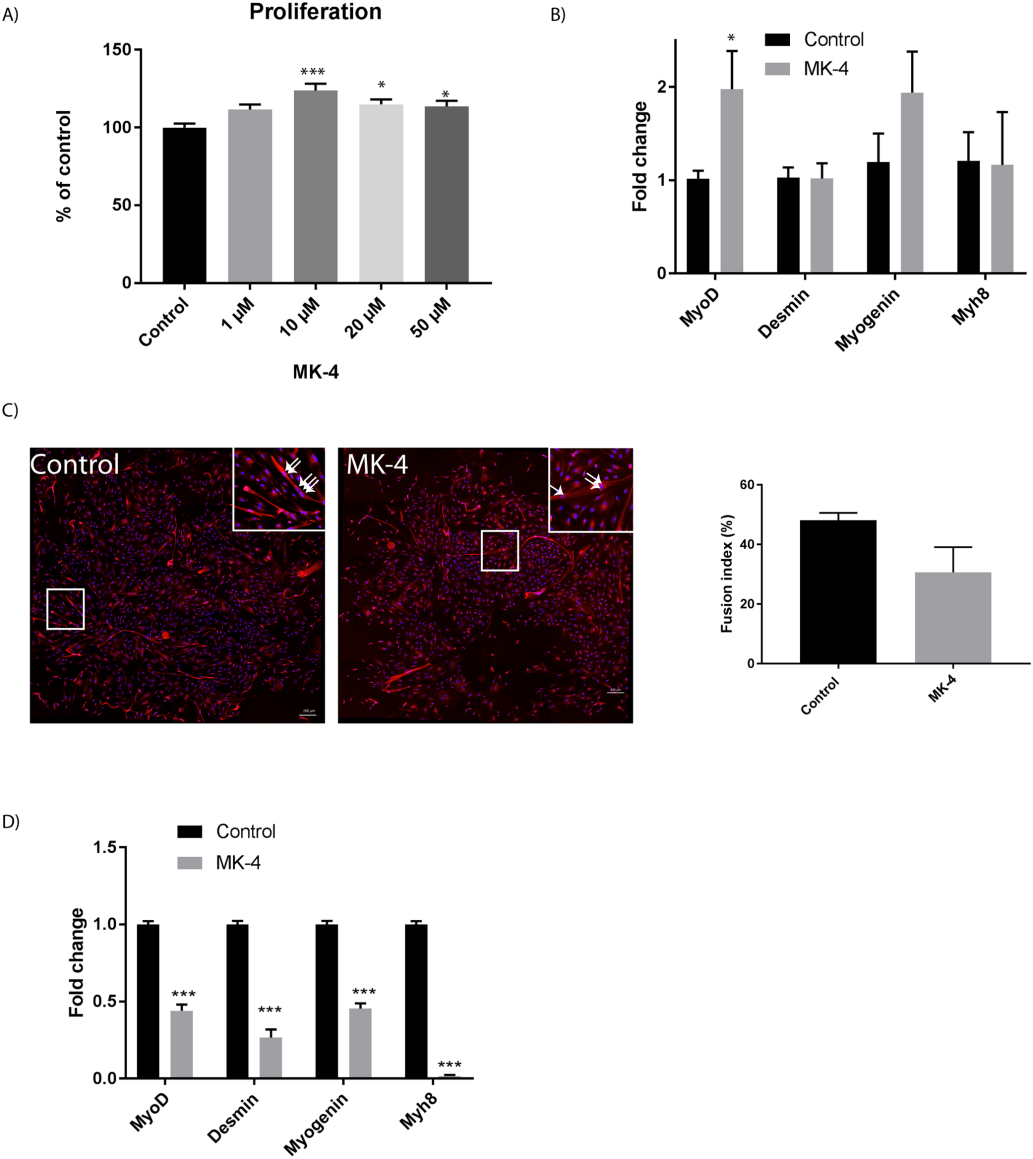
Effect of MK-4 incubation on muscle proliferation and differentiation. A) A dose response effect on muscle cell proliferation after 3 days upon MK-4 treatment, compared with control cells (vehicle EtOH only). The data is presented as the average of at least three independent cell culture experiments seeded out in triplicates, ± SEM. Asterisk indicate significant differences in treated cells compared with control cells (*p<0.05, ***p<0.001) statistics assessed by one-way ANOVA with Dunnett’s multiple comparison test. B) Relative gene expression (fold change) of muscle specific genes during 10 μM MK-4 treatment for 3 days. Bars show the relative mRNA expression (fold change) of *myoD*, *desmin*, *myogenin* and *myh8* in MK-4 cells compared to negative control cells (vehicle EtOH only) after 3 days of proliferation. The data is presented as the average of at least three independent cell culture experiments seeded out in duplicates, ± SEM. Asterisks denote significant differences (*p<0.05) between MK-4 treated cells compared to control cells assessed by un-paired two-tailed t-test. C) Fusion index was calculated based on scoring of at least four randomly chosen regions with nuclei and myotubes stained with Hoechst and desmin, as demonstrated in the left pictures. Scale bar as indicated. For each region the number of nuclei incorporated in myotubes and the total number of nuclei was scored. The fusion index (FI) was calculated as the percentage of total nuclei incorporated in myotubes, as demonstrated in the right panel. D) Relative gene expression (fold change) of muscle specific genes during 10 μM MK-4 treatment for 3 days of differentiation. Bars show the relative mRNA expression (fold change) of *myoD*, *desmin*, *myogenin* and *myh8* in MK-4 cells compared to negative control cells (vehicle EtOH only) after 3 days of differentiation. The data is presented as the average of at least three independent cell culture experiments seeded out in duplicates, ± SEM. Asterisks denote significant differences (***p<0.001) between MK-4 treated cells compared to control cells, as assessed by un-paired two-tailed t test.

Furthermore, it has been shown that after 72 hours of MK-4 treatment, there was a significant increase in muscle cell proliferation, compared to control cells (Fig 2A), with 10 μM MK-4 showing the best effect (Figs 1B and 2A). This concentration was therefore chosen in the following experiments. The transcription factor myoD is important during cell proliferation and early differentiation, while myogenin is required for the proper differentiation of most myogenic precursor cells during the process of myogenesis. Desmin and MYH8 are proteins highly expressed in differentiated cells. We therefore investigated the gene expression of *myoD*, *myogenin*, *myh8* and *desmin* in proliferating cells incubated for 3 days with 10 μM MK-4 (Fig 2B). Our results show that the *myogenin* and *myoD* gene expression increased during MK-4 treatment compared to control cells (vehicle EtOH only), the latter gene increased significantly. Interestingly, this contrasts with osteoblast cells where vitamin K2 regulate osteoblast differentiation through increased expression of osteoblast differentiation markers, but with no effect on cell proliferation [28]. Both vitamin D and K play central roles in calcium metabolism, and there is evidence of a synergistic interplay within these vitamins on bone and cardiovascular health [29]. Previous studies have demonstrated that incubation with the fat-soluble vitamin D decreases muscle cell proliferation and enhances myogenic cell differentiation [19, 30]. We therefore investigated if incubation with vitamin K had any effect on differentiating muscle cells. Complex, multinucleated myotubes were observed after 3 days in differentiation media, and interestingly the process was slower in cells incubated with 10 μM MK-4 compared with control cells, which displayed more complex and branched myotubes (Fig 2C). Likewise, when we examined the relative gene expression of muscle related markers, we observed a reduced expression of all the markers in MK-4 incubated cells compared with control cells (Fig 2D). More experiments are necessary to determine a possible interaction between vitamin D and K in skeletal muscle.

**Fig 3:**
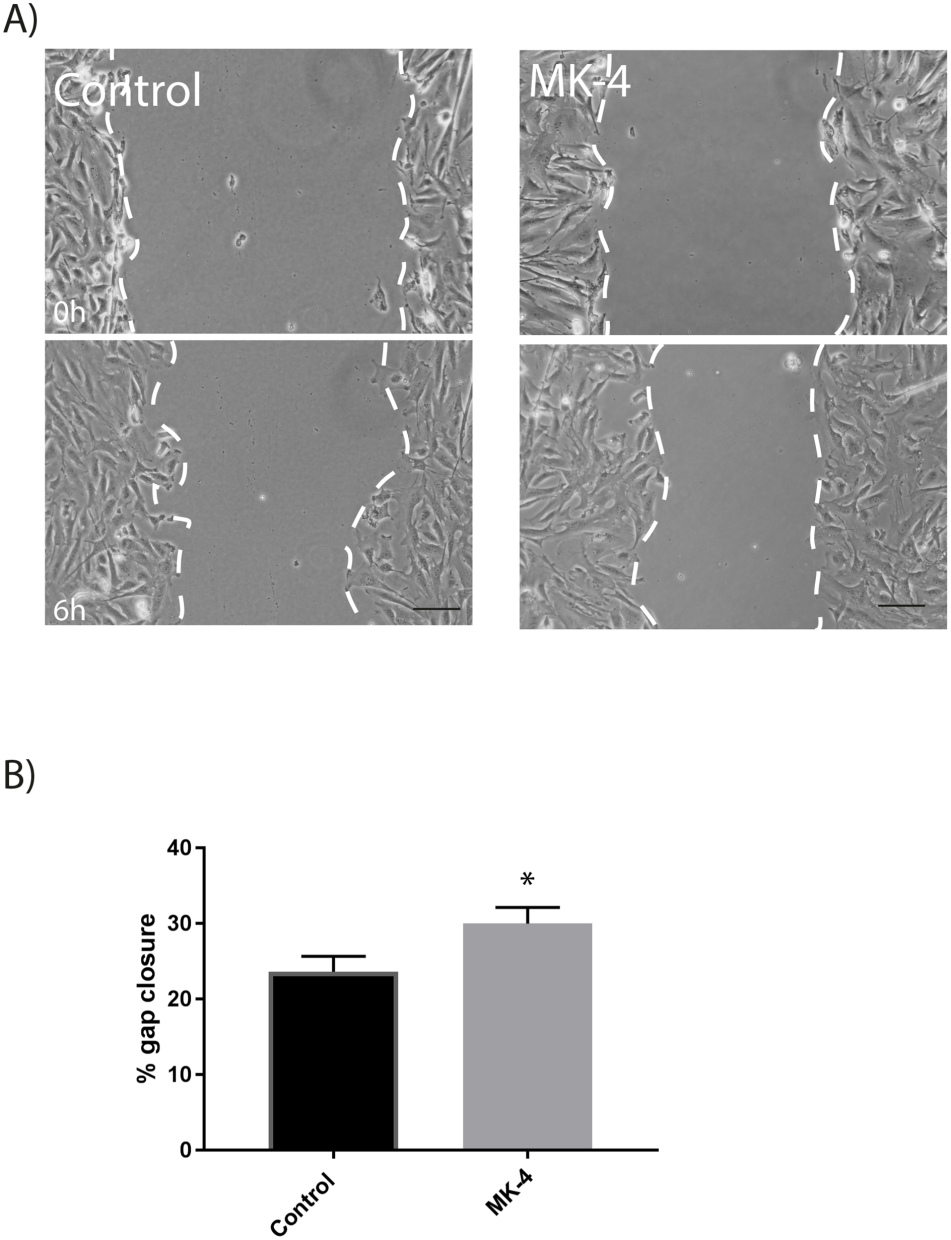
Cell migration during 10 μM MK-4 treatment, determined by a scratch wound analysis. A) Representative images of scratch wounds at 0 and 6 h after wounding in cells treated with vehicle EtOH only (control cells) or 10 μM MK-4. Scale bar 10 μm. Dotted lines define the areas lacking cells. B) Percent gap closure upon treatment, quantified based on images from A). The distance between wound edges were manually measured for ten points of the cell migration edges on each image, and the values were used to calculate the percent gap closure at 6h relative to that at 0h. The experiments were performed with at least three independent cell culture experiments, and a minimum of three images were quantified for each cell culture each experiment. Asterisk denote significant differences (* p<0.05), statistics assessed by un-paired two-tailed t test.

**Fig 4:**
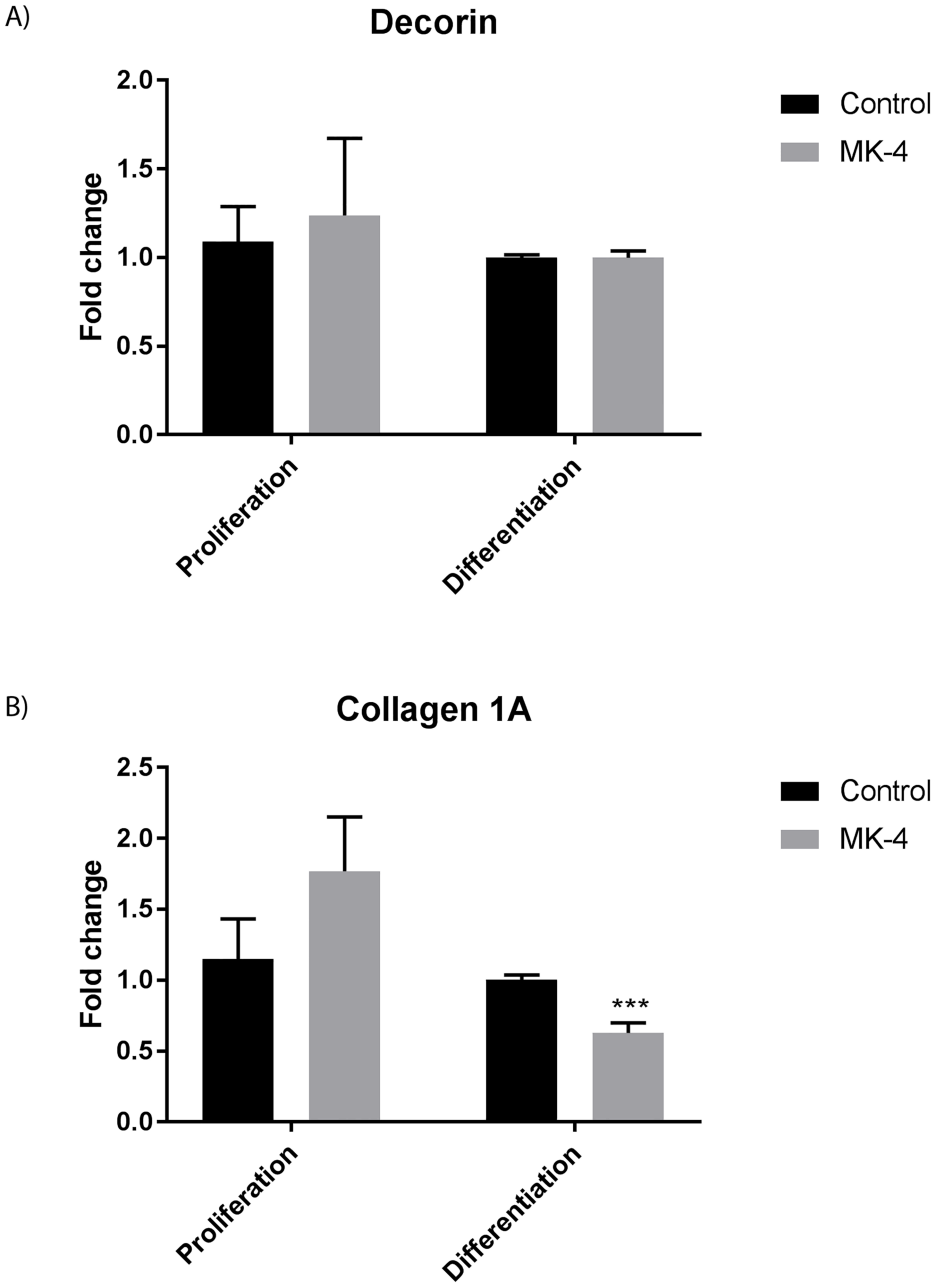
The relative difference in gene expression of extracellular matrix markers during 10 μM MK-4 treatment in proliferating and differentiating muscle cells. Relative gene expression (fold change) of extracellular matrix markers *decorin* A) and *collagen 1A* B) during 10 μM MK-4 treatment for 3 days of proliferation or 3 days of differentiation compared to negative control cells (vehicle EtOH only). The data is presented as the average of at least three independent cell culture experiments seeded out in duplicates, ± SEM. Asterisks denote significant differences (***p<0.001) between MK-4 treated cells compared to control cells, statistics assessed by un-paired two-tailed t test.

We performed a scratch wound analysis to monitor a possible effect of MK-4 on cell migration (Fig 3). Our experiments demonstrated that adding MK-4 to muscle cells significantly improved gap closure and cell migration compared to control cells. Extracellular matrix (ECM) proteins are vital for cell migration during myogenesis [15]. Cell migration is a controlled process where actin filaments are rapidly changing, leading to formation and disassembly of cell adhesion sites. External stimuli are converted to intracellular signalling through integration of integrins that bind to ECM proteins, such as collagens, fibronectin and laminin 1. Previous studies have shown that vitamin K2 is a transcriptional regulator of ECM-related genes that may contribute to collagen assembly in osteoblast cells [31]. We did not find any significant difference in the relative gene expression of *decorin* in either proliferating cells or differentiating cells (Fig 4A). This marker is previously shown to be important for differentiation and maturation of osteoblast cells [32, 33]. Recent findings suggest a potential role for collagen type I in regulating skeletal muscle cell differentiation, where experiments using C2C12 cells demonstrated that proliferating myoblasts produced collagen type 1, and this ability was lost during differentiation [34]. This is in line with our findings, with an increased expression of *collagen 1A* in proliferating cells, and a significant reduction in gene expression of *collagen 1A* in differentiating cells compared with control cells (Fig 4B).

**Fig 5:**
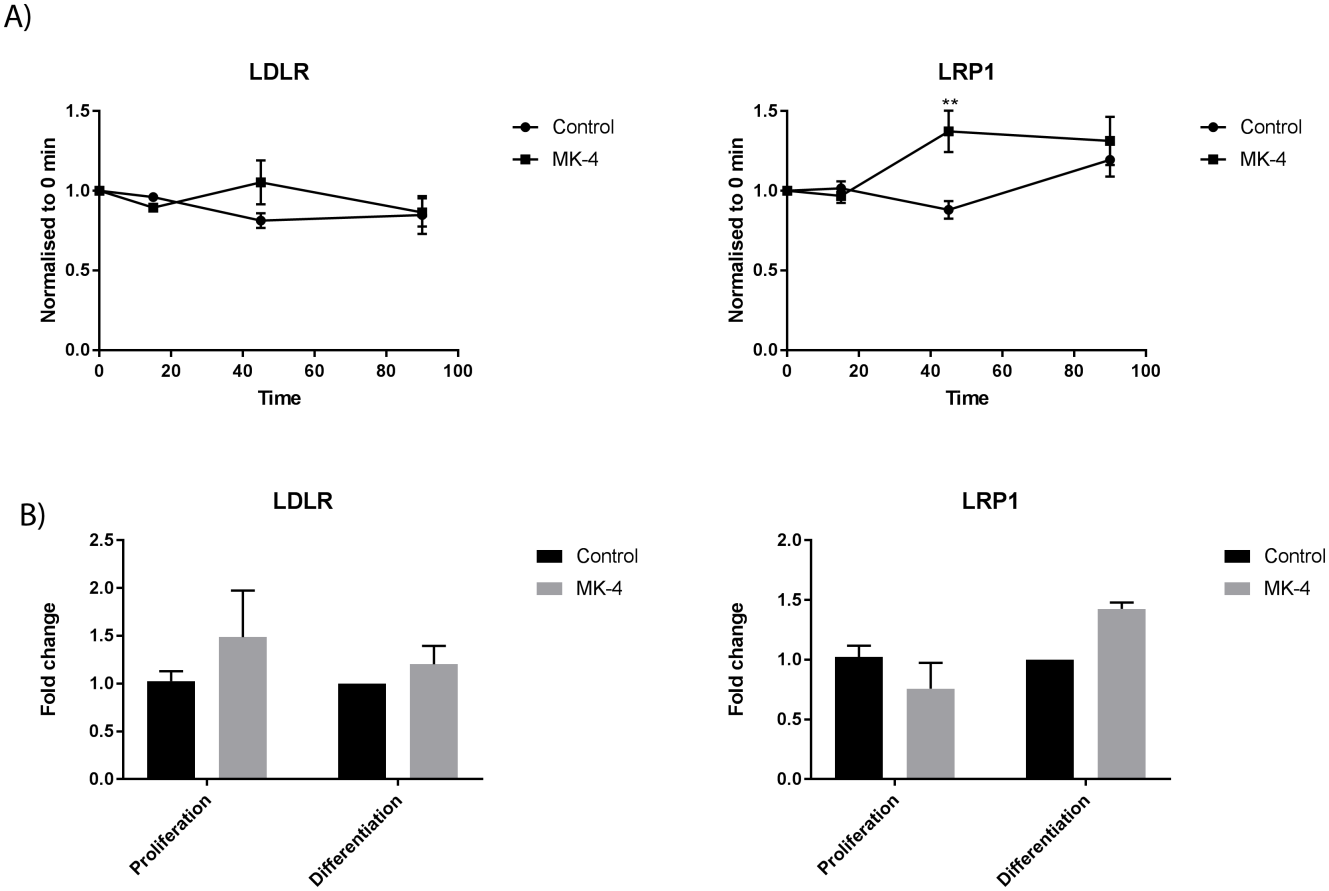
The relative difference in gene expression of LRP1 and LDLR during 10 μM MK-4 treatment. The relative mRNA expression of LRP1 and LDLR immediately after 0 min, 15 min, 45 min and 90 min stimulation A), and in proliferating and differentiating cells B), in MK-4 cells compared to negative control cells (vehicle EtOH only). The data is presented as the average of at least three independent cell culture experiments seeded out in duplicates, ± SEM. Asterisks denote significant differences (**p<0.01) in gene expression between MK-4 treated cells compared to control cells (vehicle EtOH only) at 45 min incubation, statistics assessed by un-paired two-tailed t test.

The molecular mechanism on how vitamin K is delivered to muscle cells is unknown, but previous experiments have suggested that receptor-mediated endocytosis of low-density lipoprotein receptor-related protein 1 (LRP1) and low-density lipoprotein receptor (LDLR) are important for delivery of vitamin K to bone cells [35]. Niemeier and colleagues demonstrated that LRP1 plays a significant role in vitamin K uptake in cultured osteoblasts after 20 min, as well as uptake of lipoprotein-associated vitamin K into osteoblasts in vivo, using a murine model [35, 36]. They suggest that LRP1 play a predominant role in the mechanism for delivery of lipophilic vitamins to the human bone. We show in our results that the LRP1 and LDLR gene expression peak shortly after MK-4 stimulation, suggesting these receptors to be important in MK-4 uptake in muscle cells. More experiments are necessary to further elucidate the role of LRP1 and LDLR in MK-4 uptake in muscle cells. No significant effects were observed in these receptors after MK-4 stimulation in proliferating or differentiating cells (Fig 5B),

It is well established that vitamin K has a fundamental role in blood clotting and bone homeostasis [37, 38]. However, many published studies have limitations, e.g. it is difficult to estimate the vitamin K status in humans. Vitamin K is transported by lipoproteins (mainly chylomicrons) in blood, and is usually measured in plasma [39, 40]. Hence, variations in lipoprotein profile may affect the results, and plasma levels may therefore not be sufficient to measure vitamin K status. Whether liver storage may be an indicator of vitamin K status remains to be established (see Palermo et al for systematic review of the impact of vitamin K on bone health [2]). As far as we know, there are currently no reports on vitamin K status and muscle function in humans, or studies demonstrating a beneficial effect of vitamin K in skeletal muscle. We have previously developed a model system using bovine primary skeletal muscle cells [17]. One of the advantages of this model system is that the cattle genome more closely resembles humans than rodents [41], and our experiments demonstrated that proliferating muscle cells express the biomarker NCAM similar to human muscle cells, a marker not expressed by proliferating mice muscle cells [17, 42]. Although *in vitro* cell models can never replace the use of *in vivo* animal models, the cell model can provide valuable knowledge including endogenous effect, dose-response and target organ, at low cost and brief time compared to using animal models.

We show in our study that MK-4 influences skeletal muscle cells with enhanced proliferation and cell migration *in vitro*, and reduced cell differentiation, through the involvement of the LRP1 and LDLR receptors. As such, we believe this study demonstrate the potential of vitamin K2 in maintaining normal muscle function, laying the foundation for more comprehensive future studies on the health effects of vitamin K2 in humans.

## Supporting information

S1 Fig Localization and expression of myoD and the muscle marker neural cell adhesion molecule (NCAM) after cell isolation. Proliferating cells were fixed with ice-cold EtOH, immunostained with either rabbit anti-myoD (A) or mouse anti-NCAM (B) followed by DyLight 649-conjugated donkey anti-rabbit (red) or Goat anti-mouse Alexa 488 (green) before fluorescence microscopy analysis (ZEISS Axio Observer Z1 microscope). Nuclei were stained with Hoechst (blue). Scale bar as indicated. Arrows indicate MyoD or NCAM positive cells, arrowhead indicate NCAM-negative cells.
(TIF)
