# Supplementary figures and images for: Vitamin K2 improves proliferation and migration of bovine skeletal muscle cells in vitro
Source: PLoS One. 2018 Apr 4;13(4):e0195432. doi: 10.1371/journal.pone.0195432 (PMC5884547; doi:10.1371/journal.pone.0195432)

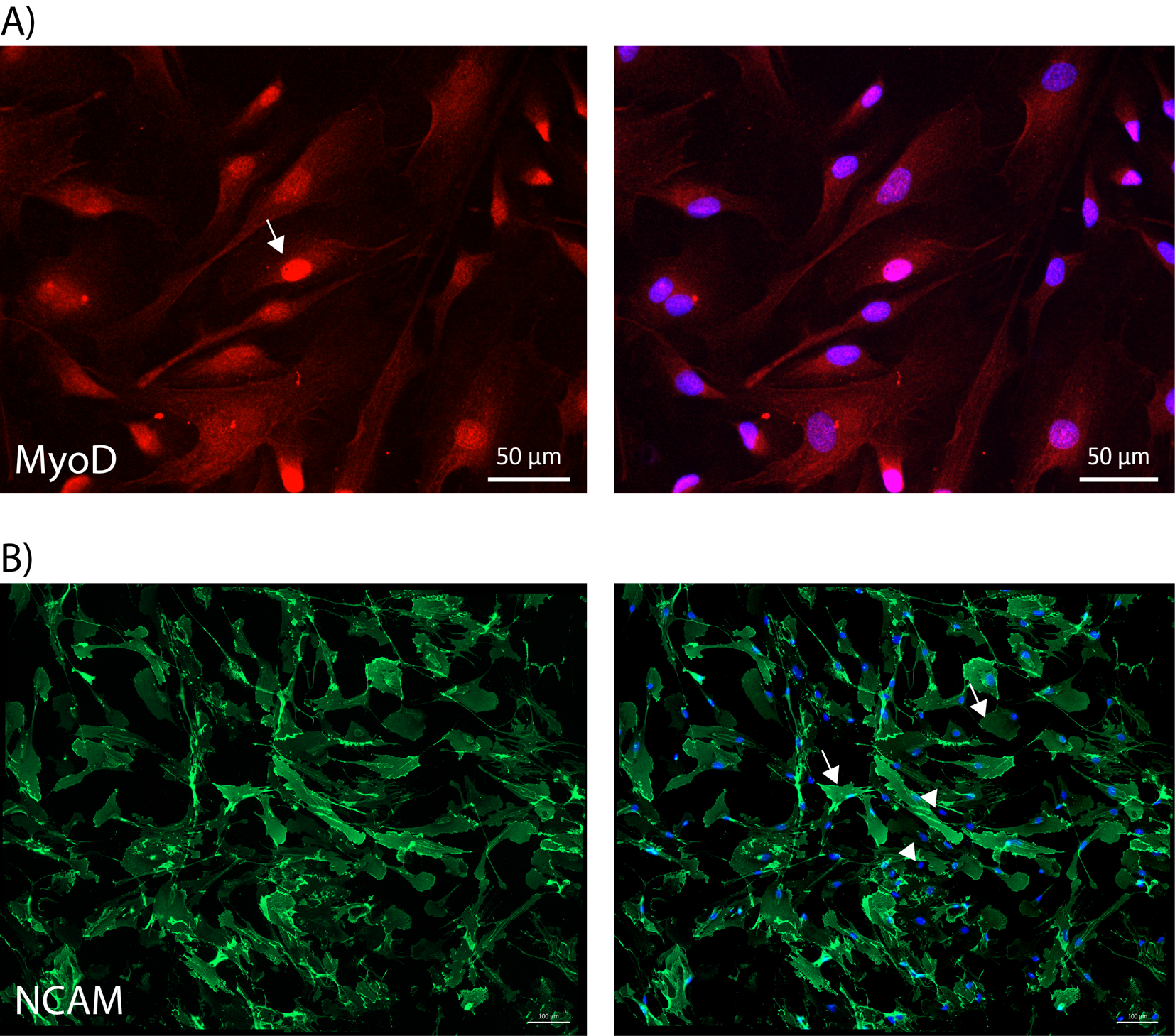

Supplement: S1 Fig — Proliferating cells were fixed with ice-cold EtOH, immunostained with either rabbit anti-myoD (A) or mouse anti-NCAM (B) followed by DyLight 649-conjugated donkey anti-rabbit (red) or Goat anti-mouse Alexa 488 (green) before fluorescence microscopy analysis (ZEISS Axio Observer Z1 microscope). Nuclei were stained with Hoechst (blue). Scale bar as indicated. Arrows indicate MyoD or NCAM positive cells, arrowhead indicate NCAM-negative cells. (TIF) [file pone.0195432.s001.tif]
